# Complete Genome Sequences of Two Central European *Brucella suis* bv. 2 Haplotype 2c Strains Isolated from Wild Boars

**DOI:** 10.1128/genomeA.00686-14

**Published:** 2014-07-10

**Authors:** Ana Cristina Ferreira, Rogério Tenreiro, Maria Inácia Corrêa de Sá, Ricardo Dias

**Affiliations:** a Instituto Nacional de Investigação Agrária e Veterinária, IP (INIAV, IP), Lisbon, Portugal; b Universidade de Lisboa, Faculdade de Ciências, Centro de Biodiversidade, Genómica Integrativa e Funcional (BioFIG), Lisbon, Portugal

## Abstract

The *Brucella suis* haplotype 2c is commonly isolated from wild boars and domestic pigs across Central Europe, though it is rarely described in the Iberian Peninsula. We report here the complete and annotated genome sequences of two haplotype 2c strains isolated from wild boars in the northeast region of Spain, above the Ebro River.

## GENOME ANNOUNCEMENT

*Brucella suis*, the causative agent of swine brucellosis, is classified in five biovars that preferentially infect different animal hosts: biovars 1 and 3 affect domestic pigs and wild boars (*Sus scrofa*), biovar 2 also affects hares (*Lepus europeaeus*), biovar 4 infects reindeer and caribou, and biovar 5 infects rodents only. In contrast to biovars 1, 3, and 4, biovar 2 has rarely been isolated from humans, and its zoonotic role is questioned (1). In Portugal and Spain, *B. suis* biovar 2 is the unique biovar isolated from domestic pigs and wild boars, and all strains share specific molecular characteristics, establishing an Iberian clone (3, 9).

*B. suis* biovar 2 haplotype 2c strains Bs364CITA and Bs396CITA were isolated from wild boars in the northeast region of Spain (above the Ebro River), biotyped according to standard bacteriological procedures (2), and characterized through PCR-restriction fragment length polymorphism (RFLP) analysis for *omp2a*, *omp2b*, and *omp31* genes and multilocus variable-number tandem-repeat (VNTR) analysis (MLVA). Both strains displayed molecular patterns distinct from those of the Iberian clone and had never been reported in Iberian domestic pigs, but they were similar to biovar 2 strains isolated from pigs and wild boars from Central European countries (3). Here, we report the complete and annotated genome sequences of these two strains.

Genomic libraries were performed using the TruSeq DNA sample preparation kit. The genomic sequences were obtained by Illumina HiSeq 2000 technology with a paired-end 35-bp protocol, generating a total of 10,021,232 and 8,358,412 high-quality reads (Phred score, >30) for Bs364CITA and Bs396CITA, respectively. The reads were *de novo* assembled using de Bruijn graph method (Velvet version 1.2.09) (5), yielding 55 (*N*_50_, 123,750) and 77 (*N*_50_, 83,863) contigs for Bs364CITA and Bs396CITA, respectively. Visual inspection of alignment of the fastq reads for each contig was performed using Tablet 1.14.04.10. Scaffolding was guided by the optical mapping method (MapSolver version 3.2; OpGen Technologies, Inc.), and gap filling was performed by PCR and the Sanger method (4). Low-coverage regions (<20×) and all indels (compared with *B. suis* ATCC 23445) were confirmed by Sanger resequencing. A total of 3,383 (Bs364CITA) and 3,387 (Bs396CITA) coding DNA sequences (CDS) were predicted and annotated through the RAST server (6). Three copies each of 5S, 16S, and 23S rRNA genes were identified using RNAmmer (7), and a set of 54 copies of tRNA genes were predicted with tRNAscan-SE 1.21 (8). The genomes have similar sizes and are composed of two circular chromosomes, with approximately 1.93 and 1.40 Mb and an overall G+C content of 57.2%. Comparative genomic analyses were performed using the genome sequences of *B. suis* ATCC 23445 (biovar 2; accession no. CP000911 and CP000912) and *B. suis* 1330 (biovar 1; accession no. AE014291 and AE014292) as references. Both strains presented 99% similarity with the reference strain of biovar 2 and 98% with *B. suis* 1330 (biovar 1).

### Nucleotide sequence accession numbers.

The complete genome sequences have been deposited in GenBank under the accession no. CP007697/CP007698 and CP007720/CP007721 for *B. suis* Bs364CITA and Bs396CITA chromosomes I/II, respectively.
